# MiR-200c and HuR in ovarian cancer

**DOI:** 10.1186/1471-2407-13-72

**Published:** 2013-02-08

**Authors:** Silvia Prislei, Enrica Martinelli, Marisa Mariani, Giuseppina Raspaglio, Steven Sieber, Gabriella Ferrandina, Shohreh Shahabi, Giovanni Scambia, Cristiano Ferlini

**Affiliations:** 1 Laboratory of Antineoplastic Pharmacology, Department of Obstetrics and Gynecology, Catholic University of the Sacred Heart, Rome, 00168, Italy; 2 Reproductive Tumor Biology Research, Biomedical Laboratory, Department of Obstetrics and Gynecology, Danbury Hospital Research Institute, 131 West Street, Danbury, CT, 06810, USA; 3 Department of Oncology, Jean Paul IInd Research Foundation, Campobasso, 86100, Italy

**Keywords:** Ovarian cancer, miR-200c, Class III beta-tubulin, HuR, Predictive biomarkers

## Abstract

**Background:**

MicroRNAs in solid malignancies can behave as predictors of either good or poor outcome. This is the case with members of the miR-200 family, which are the primary regulators of the epithelial to mesenchymal transition and have been reported to act as both oncogenes and tumor suppressors. This study assessed the role of miR-200c as regulator of class III β-tubulin (TUBB3), a factor associated with drug-resistance and poor prognosis in ovarian cancer.

**Methods:**

Expression of miR-200c was assessed in a panel of ovarian cancer cell lines with inherent or acquired drug-resistance. Stable overexpression of miR-200c was obtained in A2780 and Hey cell lines. Crosslinking-coupled affinity purification method and ribonucleic-immunoprecipitation assay were used to characterise the complexes between miR-200c, HuR and 3^′^UTR region of TUBB3 mRNA. Nanofluidic technology and immunohistochemistry were used to analyze the expression of HuR, TUBB3 and miR-200c in 220 ovarian cancer patients.

**Results:**

In a panel of ovarian adenocarcinoma cell lines, we observed a direct correlation between miR-200c expression and chemoresistance. In A2780 cells miR-200c targeted TUBB3 3^′^UTR, while a positive correlation was observed between miR-200c and TUBB3 expression in most of the other cell lines. Through the analysis of 3^′^UTR-associated complexes, we found that the miR-200c can increase the association of the RNA binding protein HuR with TUBB3 mRNA, whereas HuR binding enhanced TUBB3 mRNA translation. Most importantly, in our analysis on 220 ovarian cancer patients we observed that overexpression of miR-200c correlated with poor or good outcome depending on the cellular localization of HuR.

**Conclusion:**

This study suggests a model for the combined regulatory activity of miR-200c and HuR on TUBB3 expression in ovarian cancer. When HuR is nuclear, high expression of miR-200c inhibits TUBB3 expression and results in a good prognosis, whereas when HuR occurs in cytoplasm, the same miRNA enhances TUBB3 expression and produces a poor outcome. These findings reveal the usefulness of multidimensional analysis in the investigation of the prognostic role of miRNA expression.

## Background

About 80% of patients with ovarian cancer respond to first-line platinum/taxane chemotherapy, but the majority who experience a relapse will be refractory to further treatment [1]. Understanding the molecular events underlying relapse is essential for identifying patients at high risk of poor outcome. Several studies reported that miRNAs can act either as oncogenes or tumor suppressors [2]. In this context, expression of the miR-200 family in particular (miR-200a, miR-200b, miR-200c, miR-141, and miR-429) was linked with ovarian cancer in multiple reports with contradictory findings. In some studies, high expression levels of the miR-200 family were associated with early relapse and decreased overall survival [3-5], while in others the opposite effect of high miR-200 expression was reported [6-9]. The precise reasons underlying such contradictory findings remain unknown. The miR-200 family is known as the main suppressor of the epithelial-to-mesenchymal transition (EMT), a reversible embryonic program aberrantly activated in tumor progression and metastasis. While most carcinomas have a differentiated phenotype, ovarian cancer cells that have undergone EMT are more invasive and more aggressive [10,11]. For miR-200s to function as inhibitors of EMT seems difficult to reconcile with observations that the miR-200 family of miRNAs is overexpressed in aggressive ovarian cancers [3-5], so the designation of this family of miRNAs as drivers of biological aggressiveness and drug-resistance is a puzzle.

One of the validated targets of the miR-200 family is TUBB3 (class III β-tubulin) [12], whose overexpression has been reported in several malignancies, including ovarian cancer [13]. Posttranscriptional inhibition of TUBB3 gene expression has been reported for miR-200c in Hey ovarian cancer cells and in HeC50 endometrial cancer cells [12,14]. Forced expression of miR-200c in HeC50 cells and in a xenograft model reversed resistance to chemotherapy [12,14,15] suggesting that miR-200c is a factor protective against tumor aggressiveness and chemoresistance. We have shown previously that TUBB3 gene is conditionally expressed as an adaptive mechanism of resistance to low oxygen and glucose levels [16,17], conditions correlated with aggressiveness in cancer [18]. Interestingly, in hypoglycemic conditions, TUBB3 is induced by posttranscriptional regulation and enhanced by association with the RNA-binding protein (RBP) HuR to the 3′UTR region of the mRNA, but the role of miR-200c in this context is unknown.

The molecular mechanisms whereby HuR modulates translation are not fully clarified, although it is clear that HuR regulates numerous genes encoding proteins implicated in carcinogenesis [20]. Nevertheless, an increasing number of studies have revealed that HuR can modulate gene expression through its interplay with miRNAs. Binding of HuR may suppress the inhibitory effect of miRNAs, although HuR can also synergize miRNAs to repress gene expression [20,21].

This study aimed to clarify the role of the miR-200c as driver of biological aggressiveness in ovarian cancer. We noticed in vitro a direct correlation between expression of miR-200 family members and chemoresistance in most of the ovarian adenocarcinoma cell lines analyzed, and in particular we noted a clear correlation between miR-200c and TUBB3 expression. Using a multidimensional approach, we analyzed TUBB3 (gene and protein), HuR and miR-200c. The results suggested that the same miRNA, miR-200c, can act either as a suppressor or enhancer of the aggressive phenotype, depending upon the localization of HuR. This result offered a possible explanation for the discrepancies among the clinical reports describing miR-200c as a suppressor or enhancer of aggressiveness in solid malignancies.

## Methods

### Cell cultures and reagents

A2780, OVCAR-3, A2780-CIS, and A2780-ADR cells were purchased from the European Collection of Cell Cultures. TC1 is a clone derived from A2780 cells chronically exposed to paclitaxel [22]. OVCAR-EPO cells correspond to OVCAR-EPO10 cell line obtained from OVCAR-3 cells as patupilone-resistant, while Hey-EPO are Hey-derived patupilone resistant cells. Culture media were selected according to the suggestions of European Collection of Cell Cultures. Growth experiments and transient transfection with siHuR and siC oligonucleotide duplex were performed and analyzed as previously described [16].

A 301-bp DNA fragment including the sequence of the pre-miR-200c (NT_009759) was amplified with the primers forward 5^′^-ACAAGCTTAGGAAGTGTCCCCAGGGACTCG-3^′^ and reverse 5^′^-AACTCGAGACGCTCTCAGCTCAAGACGAGG-3^′^ and cloned in pUSE(+) expression vector (Upstate Biotechnology), obtaining the pUSE-200c plasmid. The empty pUSE vector served as control. After electroporation, cells were selected in the presence of G418 (1.5 μg/mL) and when colonies appeared cloned at limiting dilution. Twelve clones were screened and those with the highest expression chosen for further analysis.

### Real-time quantitative PCR, Western blotting and Immunohistochemistry

MiRNAs reverse transcription and PCR reactions were performed on Trizol (Invitrogen, Carlsbad, CA, USA) isolated total RNAs using TaqMan MicroRNA Assays kit (Applied Biosystems, Foster City, CA, USA). Quantitative PCR on mRNAs was performed as previously described [16]. Western blots were done on total lysates or on nuclear/cytoplasmic fractions as previously described [16], with the following antibodies: anti-human TUBB3 polyclonal (1:1000, Covance, Princeton Township, NJ), anti-HuR (1:500, Santa Cruz, Santa Cruz, CA), anti-β-actin (1:5000, Sigma, Saint Louis, MO), anti-SNRP70 (1:1000, Abcam, Cambridge, UK), anti-GAPDH (1:5000, Abcam, Cambridge, UK). Blots were visualized by enhanced chemiluminescence procedures (Amersham, GE-Healthcare, Buckinghamshire, UK) as described by the manufacturers. The expression of HuR and TUBB3 was immunohistochemically assessed in a series of 220 ovarian cancers. Immunostaining for HuR was performed as previously described [16]. For the analysis of the expression of TUBB3, antigen retrieval procedure was performed by microwave oven heating in 10mM citric acid, pH 6.0 (2 times for 4 min.). TUBB3 protein was identified after overnight incubation at 4°C by using the monoclonal antihuman antibody (clone TUJ1;1:300; Covance) in 20% normal goat serum. The En Vision-mouse+ System-HRP (DAKO, Carpinteria, CA, USA) was used. Diaminobenzidine was used as a chromogen (DAB substrate System, DAKO). Sections were counterstained with haematoxylin.

### Luciferase assays

The 292-bp 3^′^UTR sequence of the TUBB3 gene (NM_006086) was cloned in the XbaI site of pGL3-Promoter Vector (Promega, Madison, WI), downstream of the firefly luciferase coding region, obtaining the pGL3-TUBB3-UTR construct. pGL3-TUBB3-UTRm vector was derived by inverse PCR on pGL3-TUBB3-UTR plasmid, resulting in a 10-bp deletion (GCAGTATTTA) which includes the seed sequence for miR-200c. The control vector pGL3-V was obtained cloning a 445-bp sequence from the MCS of pBluescript SK (nt 977–532) in the XbaI site of pGL3-Promoter Vector. A2780 cells were transfected with the described reporter vectors together with the renilla luciferase normalization plasmid (pRL-TK), using Transfectin (Bio-Rad, Hercules, CA). Cells were harvested 48 hours later for analysis using Dual Luciferase Reporter assay system (Promega, Madison, WI).

### Purification of S1-tagged mRNPs and RIP assay

The UTR-S1 vector was obtained inserting in pUSE(+) expression vector (Upstate Biotechnology, Lake Placid, NY) the following sequences: 1) the coding sequence of the firefly luciferase and the TUBB3 3^′^UTR region, amplified from the described pGL3-UTR construct; 2) the linker sequence L1 amplified from pBluescript SK vector (nt 701–825); 3) the S1 aptamer sequence, obtained by annealing the primers tag-S1-F 5^′^-AATTCACCGACCAGAATCATGCAAGTGCGTAAGATAGTCGCGGGCC-3^′^ and tag-S1-R 5^′^-GGCCGCCCCGGCCCGCGACTATCTTACGCACTTGCATGATTCTGGT-3^′^. The UTRm-S1 vector differs from UTR-S1 for the mutation already described in pGL3-UTRm construct. The S1-tagged reporter constructs were stably transfected in A2780 cell line as described above and the expression of the exogenous mRNA was quantified by Q-PCR with primers LUC-F1 5^′^-CTTACTGGGACGAAGACGAACAC-3^′^ and LUC-R1 5^′^-GGGAAGACCTGCGACACCTG-3^′^. The purification of the S1-tagged mRNA/RBP was performed on cells treated with glucose-free medium for 48 hours, then incubated with 0.2% formaldehyde for the cross-linking and processed as described-by Vasudevan & Steitz [23]. Western blot were performed with the antibody anti-Ago2 (1:250, Abcam, Cambridge, UK) and anti-HuR (1:500, Santa Cruz, Santa Cruz, CA). RIP was performed as described [16], with the modification of treating the cells in 0.2% formaldehyde before the harvesting, as above described for purification of S1-tagged mRNA/RBPs.

### Nanofluidic analysis of micro-RNA and gene expression

FFPE samples were obtained from ovarian cancer that had been preserved between 2000 and 2008 following the approved Danbury Hospital Internal Review Board protocol. FFPE samples were cut to 10 μm thickness and two tissue slices were put into a 1.5 ml tube. One milliliter of xylene was added for deparaffinization followed by mixing twice with a high speed vortex for 3 min at room temperature. Total RNA was then automatically extracted with the QIAcube using the Qiagen miRNeasy FFPE kit (Valencia, CA) following manufacturers’ protocols. The RNA from the cell line A2780 was automatically extracted with the QIAcube using the Qiagen miRNeasy kit (Valencia, CA) following manufacturer’s protocols. RNA quantity and the quality were assessed by Agilent 2100 Bioanalyzer (Agilent Technologies, Santa Clara, CA). Analysis was carried out using the 48.48 dynamic array (Fluidigm Corporation, CA, USA) and a Biomark platform following the manufacturer’s protocol.

### Statistical analysis

Overall survival (OS) and progression free survival (PFS) were calculated from the date of diagnosis to the date of progression/death or date last seen. Medians and life tables were computed using the product-limit estimate by the Kaplan-Meier method and the Wilcoxon test was employed only to assess statistical significance. Multivariate analysis assessed the clinical role of TUBB3, miR-200c, HuR pattern of staining in a model including additional significant variables in univariate analysis such as (age, stage and histotype) using the Cox proportional hazards model and nonparametric testing with the Kruskal Wallis test. *T*-test served to test differences of expression among different cells/conditions. A P value<0.05 was considered significant. Statistical analysis was carried out using JMP9 (SAS).

## Results

### Expression of the miR-200c in drug-resistant ovarian adenocarcinoma cell lines

**Figure 1:**
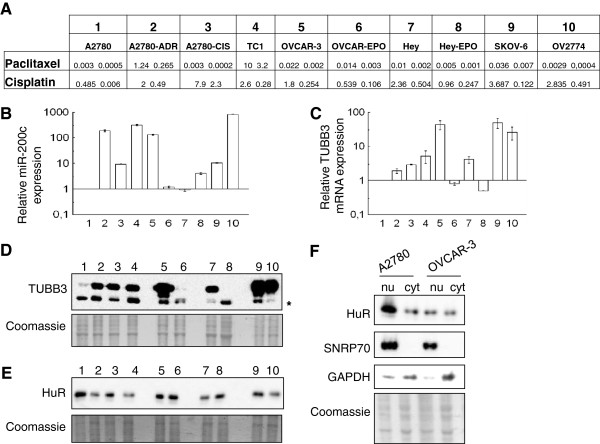
**A: Table reporting the IC50 in μmol/L for paclitaxel and cisplatin in the used panel of ovarian cancer cell lines.** The cell lines are numbered progressively and the same numbers are used to indicate the lane in which each cell line was analyzed. **B** and **C**: Analysis of miR-200c and TUBB3 mRNA expression, respectively, in the cell lines numbered as in panel A. Bars and error bars refer to mean and SD of two experiments performed in triplicate. In bar charts expression was normalized on the level measured in A2780 cells (=1). **D** and **E**: Representative Western Blot for the expression of TUBB3 and HuR, respectively,in the cell lines numbered as in panel A. The Coomassie is used as loading control. The asterisk indicated the mitochondrial form of TUBB3. **F**: Western blot analysis of HuR expression on A2780 and OVCAR-3 nuclear (nu) and cytoplasmic (cyt) fractions. SNRP70 and GAPDH were utilized as markers of nuclear and cytoplasmic extracts respectively. The protein content were used as loading control.

Expression of miR-200c was assessed in a panel of ovarian cancer cell lines with differing sensitivities to paclitaxel and cisplatin (Figure 1A). A2780 cells are markedly more drug-sensitive than OVCAR-3, Hey, SKOV-6, and OV2774 cell lines, while its derivatives A2780-ADR, A2780-CIS, and A2780-TC1 show acquired chemoresistance to different extents. OVCAR-EPO and Hey-EPO cells were obtained from OVCAR-3 and Hey cells, respectively, and show resistance to patupilone treatment but a heightened sensitivity to paclitaxel and cisplatin, compared to the parental cell lines [24,25].

Q-PCR analysis of the miR-200c in the aforementioned cell lines revealed that this miRNA exhibited a higher expression with respect to the drug-sensitive isogenic cells, regardless of whether resistance to paclitaxel and cisplatin was inherent or acquired (Figure 1B). The only exception was the expression in the Hey cell line, which was approximately the same as in A2780 cells, while miR-200c expression was fourfold higher in Hey-EPO cells.

### Correlation between miR-200c and TUBB3 expression in drug-resistant cell lines

Because TUBB3 is among the factors associated with drug-resistance in ovarian cancer, we set out to investigate the correlation between TUBB3 expression and expression of miR-200c in the same panel of cell lines. The highest TUBB3 mRNA and protein expression is observed in cell lines with native chemoresistance (OVCAR-3, Hey, SKOV-6, and OV2774 cells), but a marked increase in expression is also evident in A2780-derived cell lines with acquired drug-resistance (Figure 1C-D). OVCAR-EPO and Hey-EPO cells showed decreased TUBB3 expression compared with parental cells, as we reported previously, since epothilones seemed capable of selectively killing off those cells with high levels of TUBB3. Comparisons between TUBB3 and miR-200c expression revealed a direct correlation (R=0.49, *p*=0.03) in the majority of the cells analyzed. The only exception was in Hey and Hey-EPO cells, where an inverse correlation was observed between TUBB3 and miR-200c expression.

Since HuR is involved in the control of TUBB3 translation [16], HuR expression was analyzed in the same panel of cells. The total amount of HuR protein did not show evident correlation with TUBB3 expression in normoglycemia (Figure 1E). Nevertheless, the nuclear/cytoplasmic ratio of HuR in the different cell lines was different in normoglycemia, with A2780 exhibiting a prevalent nuclear HuR localization, while in OVCAR-3 the localization was mostly in the cytoplasm (Figure 1F).

**Figure 2:**
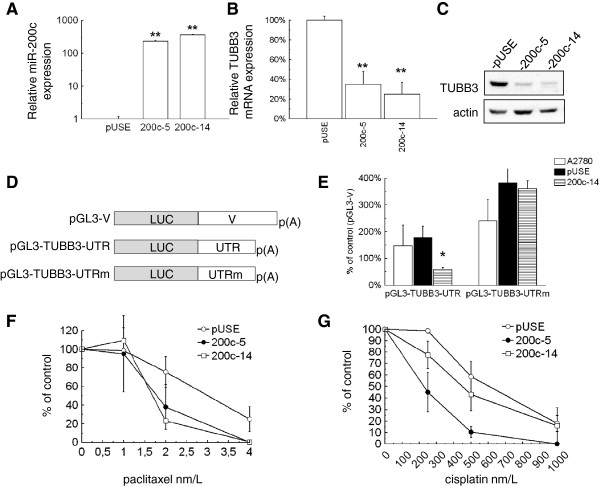
**A: Expression analysis of miR-200c in A2780 cells transformed with the empty vector (pUSE) and in two clones overexpressing the miR-200c (200c-5 and 200c-14).B**: Bar chart of TUBB3 mRNA expression obtained in the same cells. Bars and error bars refer to mean and SD of two experiments performed in triplicate. It is clearly visible that overexpression of miR-200c inhibits expression of TUBB3 gene in a significant way (double asterisks= P<0.001). The same phenomenon is evident in the western blot (**C**) showing the TUBB3 expression at the protein level. Actin served as a loading control. **D**: Schematic representation of the constructs utilized for the luciferase assays. **E**: Luciferase assays on A2780 cells transfected with the reporter vectors pGL3-TUBB3-UTR, pGL3-TUBB3-UTRm or the control pGL3-V. The relative luciferase units were calculated normalizing the firefly values for the renilla values. A clear inhibition is noticeable when pGL3-TUBB3-UTR is transfected in miR-200c is overexpressing cells . **F**-**G**: Line charts reporting dose–response curves obtained in A2780-pUSE, 200c-5 and 200c-14 cells treated with paclitaxel (F) and cisplatinum (G). Colonies were counted 14 d after drug treatment.

Recent studies demonstrated that TUBB3 expression is indeed modulated by miR-200c in Hey cells and in Hec50 endometrial cancer cells [12,14]. In order to verify this effect in A2780 cells, two cell clones overexpressing miR-200c were generated from A2780 cells. As compared with the cells transformed with the empty vector (pUSE), the 200c-5 and 200c-14 clones exhibited more than two-hundred fold miR-200c expression (Figure 2A). The 200c-5 and 200c-14 clones exhibited also a remarkable significant inhibition of TUBB3 at the gene and protein level (Figure 2B-C). A luciferase-based assay was performed to investigate the direct activity of miR-200c on TUBB3 regulation. TUBB3 3^′^UTR was cloned downstream of the firefly luciferase gene in a reporter vector (pGL3-TUBB3-UTR, Figure 2D) and mutagenized into the predicted interaction site (pGL3-TUBB3-UTRm vector). A plasmid carrying a sequence from the pBluescript vector was prepared as control (pGL3-V construct). The reporter assay experiment was performed in parental A2780, pUSE and the 200c-14 clone with the highest expression of miR-200c. The pRL-TK vector served to normalize firefly luciferase expression. Luciferase expression driven by pGL3-UTRm was 1.6-fold higher as compared with pGL3-UTR in A2780 cells, demonstrating that the predicted site of interaction with miR-200c was indeed recognized by a negative regulator (Figure 2E). Overexpression of miR-200c caused a significant inhibition of the reporter activity, while this phenomenon disappeared when the mutated pGL3-TUBB3-UTRm vector was used (Figure 2E). These findings demonstrated that miR-200c is a repressor of TUBB3 expression in A2780 cells through an interaction in the 3^′^UTR of the gene, as described previously by Cochrane and colleagues in Hey cells [12]. To investigate the possible role of miR-200c in drug resistance, clonogenic and growth inhibition assays were performed on clones that overexpressed miR-200c. Paclitaxel and cisplatin exhibited an increased activity in both clones as compared to control cells (Figure 2F-G). As an additional parameter for gauging cell proliferation, the total colony area was measured and a decrease was noted in the miR-200c overexpressing cells treated with paclitaxel and cisplatin (data not shown). These results are in line with previous evidences that TUBB3 downregulation is responsible for increased sensitivity to cisplatin and paclitaxel [24,26].

**Figure 3:**
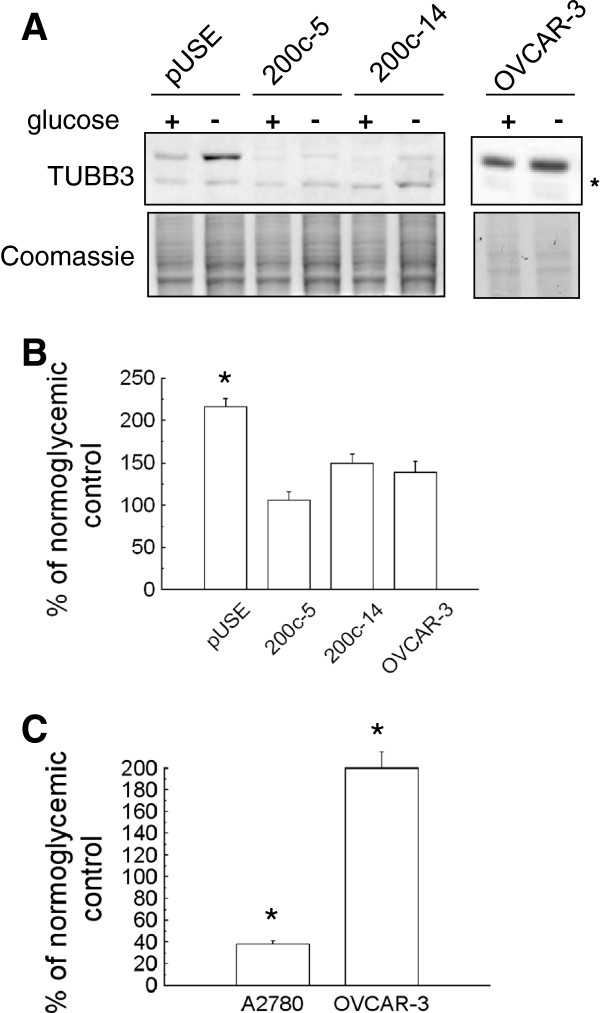
**A: Representative western blot analysis of TUBB3 expression on A2780 miR-200c overexpressing clones (200c-5 and 200c-14) relative to A2780 transfected with the empty vector (A2780-pUSE) upon treatment in hypoglycemia for 72 hours (left panels).** The same treatment was also performed on OVCAR-3 cells (right panels). The total protein content was used as loading control. The asterisk indicated the mitochondrial form of TUBB3. **B**: Densitometric analysis of the bands of panel A. Values are expressed as percentage relative to the untreated controls. Bar and error bars represent the mean and SD of two independent experiments. Only in PUSE there was an increase in hypoglycemia of TUBB3 expression which was significant. **C**: qPCR analysis of miR-200c expression on A2780 and OVCAR-3 cells as indicated, upon treatment in hypoglycemia, conditions for hours. Values are expressed as percentage relative to the untreated controls. In the two cellular models the opposite significant (asterisk=P-value <0.05) trend for miR-200c expression was noticed.

### Hypoglycemia and miR-200c control of TUBB3 expression

TUBB3 expression is induced by hypoxia and hypoglycemia, conferring increased drug-resistance on A2780 cells [16,17].

To analyze the possible involvement of miR-200c in stress-induced TUBB3 expression, A2780 miR-200c overexpressing cells were cultured in hypoglycemic conditions for 72 hours, after which time the TUBB3 protein level was assessed (Figure 3A-B). While TUBB3 protein induction was evident in control pUSE cells, a just detectable increase was noticed in miR-200c overexpressing cells, suggesting that miR-200c was capable to inhibit the overexpression of TUBB3 gene under stressing conditions.

**Figure 4:**
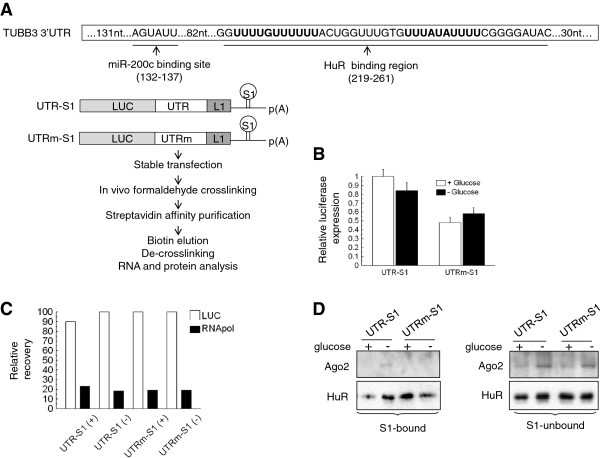
**A: The scheme above represented the TUBB3 30UTR region, with the miR-200c binding site and the HuR binding region, according to[**[12,27]**].** The schematic representations of the S1-tagged constructs were shown below, as the method utilized for the purification of the mRNP complexes. **B**: qPCR analysis of the luciferase expression in A2780 cells stably transfected with UTR-S1 or UTRm-S1 vectors and cultured in hypoglycemia for 48 hours (− glucose), in comparison with the expression in normoglycemia (+ glucose). **C**: qPCR analysis of the exogenous mRNAs recovery (LUC), upon mRNA/RBP complexes purification as indicated in panel A. A2780 cells expressing either UTR-S1 or UTRm-S1 vectors were cultured in hypoglycemia (−) or in normoglycemia (+) for 48 hours. The values are expressed as percentage relative to input mRNAs. As control also the recovery of RNA polymerase II mRNA was analyzed (RNApol). **D**: Representative Western blot analysis of Ago2 and HuR proteins in streptavidin-bound complexes (S1-bound) and in extracts not bound to streptavidin (S1-unbound) purified from cell lines as in panel C.

TUBB3 protein expression was also analyzed in the OVCAR-3 cell line, featured with high levels of TUBB3 and miR-200c (see Figure 1B-D). In this model, hypoglycemia induced a faint not significant increase (Figure 3A-B). This finding suggested that TUBB3 expression cannot be increased over a certain level in this cell line, at least in the conditions analyzed.

Endogenous miR-200c expression was assayed in A2780 and OVCAR-3 cells cultured in hypoglycemic conditions (Figure 3C). In A2780 cells miR-200c expression decreased, while in the same conditions it increased in OVCAR-3. Overall, these results suggested that A2780 and OVCAR-3 cell lines represent two different models, and prompted us to investigate the mechanism of miR-200c in regulation of TUBB3 expression, analyzing the involvement of additional factors.

### Analysis of the interaction of miRNA-200c and HuR in modulation of TUBB3 expression

We reported previously that HuR mediates increased translation of TUBB3 through the 3^′^UTR region of TUBB3 mRNA [16]. Since specific mRNAs can be relieved of miRNA-mediated suppression by HuR [20,21,28], we explored the possible interactions between HuR and miR-200c in modulating TUBB3 mRNA. The study of mRNA/protein complexes in vivo is often hampered by the rapidity of the interactions and by nonspecific binding of abundant RBPs. To overcome these limitations we set up an in vivo cross-linking–coupled RNA/RBP purification assay. We obtained a vector in which the TUBB3 3^′^UTR region was followed by an S1 sequence, an RNA aptamer which binds the streptavidin (UTR-S1, Figure 4A), along with the UTRm-S1 vector, in which the tagged 3^′^UTR sequence featured a mutation at the miR-200c interaction site (Figure 4A). After the stable transfection in A2780 cells and the treatment of the cells in hypoglycemic conditions, we checked that the expression of exogenous mRNAs was similar in A2780-UTR-S1 and A2780-UTRm-S1 cell lines in normoglycemia, as well as upon hypoglycemic treatment (Figure 4B). The analysis of specific RNAs and RNA-binding proteins associated in the S1-complexes was performed. We verified that the recovery of the exogenous mRNAs was similar in A2780-UTR-S1 and A2780-UTRm-S1 cell lines in normoglycemia, as well as upon hypoglycemic treatment (Figure 4C). The RNA polymerase II mRNA, analyzed as a negative control, was indeed detected at a much lower rate in the complexes (Figure 4C).

Western blot analysis revealed that HuR binding on UTR-S1 mRNA increased with hypoglycemic conditions, whereas binding on UTRm-S1 mRNA decreased under these conditions (Figure 4D). Ago2 binding was detectable in hypoglycemic conditions on UTR-S1 mRNA, but not on UTRm-S1 mRNA (Figure 4D). The S1-unbound samples were utilized as controls. MiR-200c was needed for the interaction of Ago2 with the TUBB3 3^′^UTR, since the deletion of its seed sequence prevented the association of any RISC containing Ago2, suggesting that miR-200c is the main miRNA interacting with TUBB3 3^′^UTR. What is more, these data support the notion that miR-200c interacted with the TUBB3 3^′^UTR in hypoglycemia and that such an interaction was important for HuR binding and increased expression of TUBB3 under stressing conditions. In this context, and at variance previous reports [14], miR-200c seemed to be capable of acting as a positive regulator of TUBB3 expression and as a factor determining biological aggressiveness.

**Figure 5:**
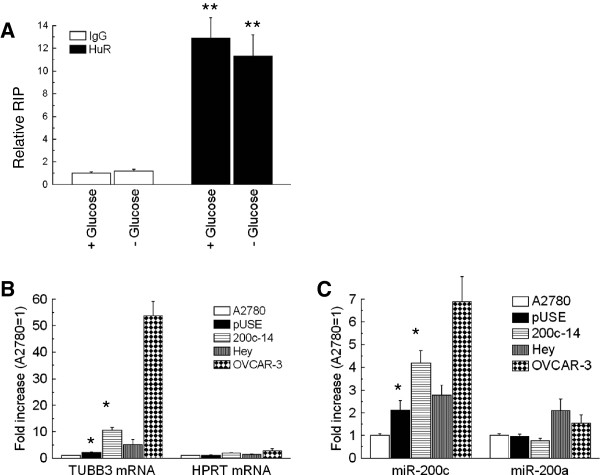
**A: Bar chart of ribonucleoprotein-immunoprecipitation (RIP) performed on A2780 cells in normoglycemia and after 48 hours of hypoglycemia with anti-HuR antibody and with an irrelevant anti IgG antibody.** The qPCR analysis of miR-200c present in the RIP complexes was quantified by normalization with the corresponding not immunoprecipitated extracts. Data were shown as relative RIP, as IgG (+) =1. **B-C**: Bar charts of RIP performed in A2780, A2780-pUSE, A2780-200c-14, Hey and OVCAR-3 cells as indicated with anti-HuR antibody and with an irrelevant anti IgG antibody. Statistical comparison was performed only among isogenic cells. Asterisk mark the significant differences as compared to A2780 parental cells while § marks the difference as compared to pUSE cells. For both significance was set at a P<0.05. The qPCR analysis of TUBB3 mRNA, HPRT mRNA, miR-200c and miR-200a present in the RIP complexes was quantified by normalization with anti-IgG RIP and the corresponding not immunoprecipitated extracts. Values were normalized for the levels obtained in A2780 cells (=1).

To further demonstrate the presence of a complex involving HuR, TUBB3 mRNA and miR-200c, ribonucleoprotein-immunoprecipitation (RIP) with anti-HuR antibody was performed on A2780 cells treated in hypoglycemic conditions. As normalized for the values detectable in the unbound fraction, there was a specific enrichment of miR-200c in the HuR-complexes in A2780 cells (Figure 5A). The RIP assay was also performed in A2780 control cells (A2780-pUSE) in the A2780-200c-14 clone, in Hey and OVCAR-3 cells (Figure 5B-D). All these cells are featured by diverse levels of miR-200c, as previously shown (Figure 1B and 2A). The recovery of TUBB3 mRNA associated with HuR was dramatically enhanced when the levels of miR-200c were higher, as it occurs in 200c-14 clone and in OVCAR-3 cells (Figure 5B). As control, also the level of HPRT in the HuR-complexes was measured. The miR-200c analysis of RIP complexes revealed that there was an increased recovery of miR-200c in OVCAR-3 and 200c-14 cells, while such phenomenon was not observed for miR-200a, utilized as control (Figure 5C). Overall, the obtained results strongly suggested the interplay of miR-200c complex and HuR in regulation of TUBB3 expression. The same strategy adopting the luciferase reporter assay was also attempted in OVCAR-3 and OV2774 cells in the presence of HuR silencing. The expression of the artificial reporter was barely detectable in multiple experiments, likely for the competition of the high expression of the natural TUBB3 gene (data not shown).

**Figure 6:**
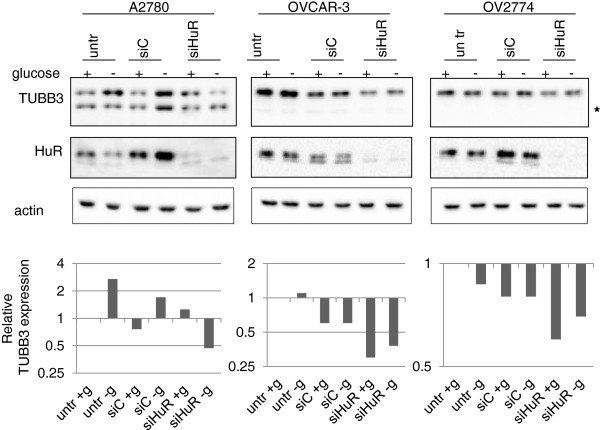
**Western blot analysis of TUBB3 and HuR expression on A2780, OVCAR-3 and OV2774 cells untrasfected (“untr” lanes) or transfected with siC and siHuR, as indicated.** Cells were cultured in normoglycemia (+) or in hypoglycemia (−) for 48 hours (for methods details, see [16]). The asterisk indicated the mitochondrial form of TUBB3. Actin served as loading control. Densitometric analysis of the TUBB3 bands corresponding to the TUBB3 cytoplasmatic form is shown on the bottom side of the panels and values are expressed as percentage relative to the untreated controls.

The relevance of HuR in the control of TUBB3 translation, already reported in the A2780 cells [16], was studied also in the different models of OVCAR-3 and OV2774 cells (Figure 6). The HuR silencing by transient transfection in A2780 cells reverted the induction of TUBB3 expression in hypoglycemia, while in the OVCAR-3 and OV2774 cells the decrease of TUBB3 expression is detectable also in basal conditions. These results confirmed the significance of this factor in the modulation of TUBB3 expression also in these models.

### Analysis of HuR, TUBB3, and miR-200c expression in 220 ovarian cancer patients

**Figure 7:**
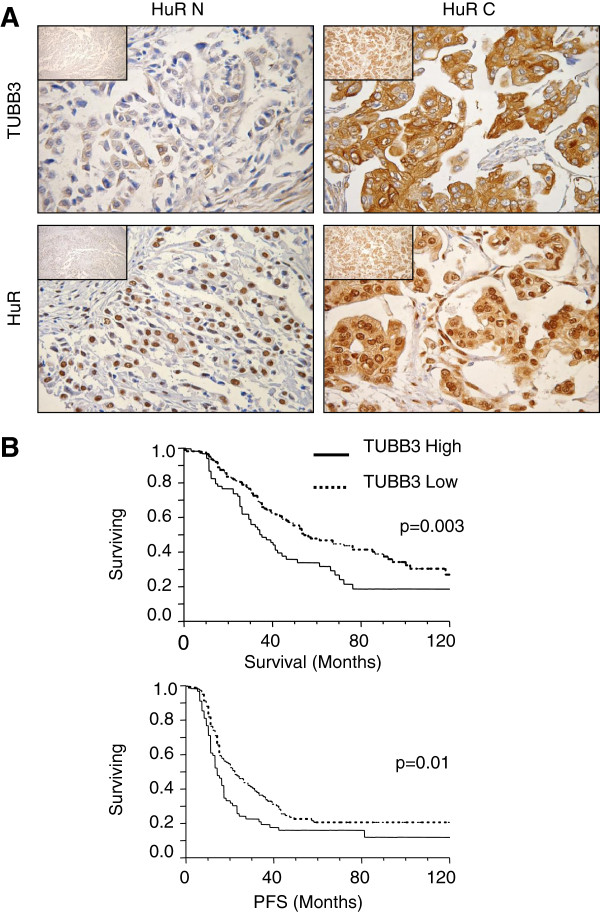
**A: Representative immunohistochemistry for HuR (upper panels) and TUBB3 (lower panels) on ovarian cancer samples**. The panels on the left represent an ovarian cancer with a nuclear staining pattern for HuR (Hur N, see text) and a faint staining of TUBB3; the panels on the right represent a sample with a nuclear and cytoplasmic staining pattern for HuR (Hur C) and a marked staining of TUBB3 (magnification 400X). In insets, lower magnification is shown. **B**: Kaplan Meier analysis of overall survival (top) and progression free survival (bottom) for ovarian cancer patients according to TUBB3 protein expression. P values are added when the differences between the two curves are significant by Wilcoxon test.

**Figure 8:**
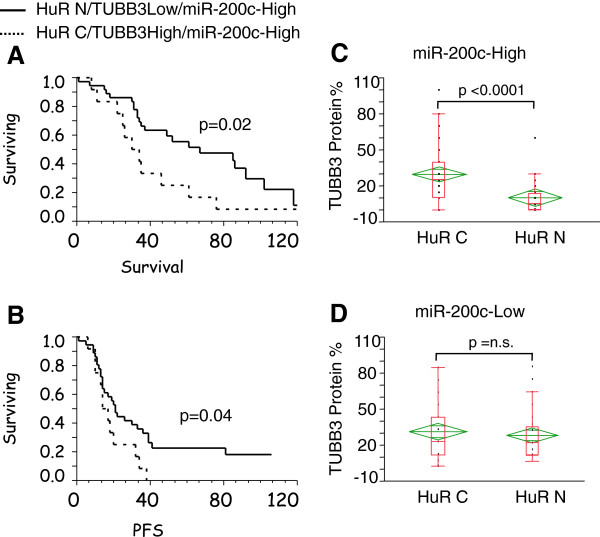
**(A-B) Kaplan Meier Analysis of overall survival (A) and progression free survival (B) for ovarian cancer patients according to HuR pattern/TUBB3 protein expression/miR-200c expression.** P values are added when the differences between the two curves are significant by Wilcoxon test. (**C**-**D**) Box-plot representing the TUBB3 protein expression according to HuR pattern of staining in patients expressing high levels of miR-200c (**C**) and low levels of miR-200c (**D**). Lines represent the range of the expression while diamond plots represent the mean and the DS interval. Data show that patients with high expression of miR-200c have significant differences in TUBB3 protein expression according to HuR pattern of staining.

A retrospective analysis was carried out in 220 ovarian cancer patients to assess the clinical relevance of our findings. Clinical features of this study subset are summarized in Table 1. Analysis of miR-200c and TUBB3 gene expression was performed using a nanofluidic genetic analyzer and a 48.48 chip array. As control miRNAs, RNU44 and MAMMU6 were used, while for TUBB3 assessment, housekeeping was performed by GAPDH. All results were normalized with expression levels measured in A2780 cells (=1). As reported previously [13], the median served as the cut-off value to identify groups of patients negative and positive for miR-200c and TUBB3 mRNA expression. HuR and TUBB3 staining was performed using immunohistochemistry (Figure 7A). A certified pathologist scored the samples as bearing only nuclear (HuR N) or both a nuclear and cytoplasmic pattern of staining (HuR C) without any prior knowledge of clinical or biological results. Similarly, the pathologist scored TUBB3 protein expression and patients were categorized as demonstrating high or low TUBB3 expression according to the percentage of positive cells, if above or below the median 20%, respectively. Of the 220 patients analyzed, 186 patients expressed detectable amounts of all 3 factors (HuR, TUBB3, and miR-200c). In keeping with our previous findings [13], TUBB3 protein expression alone was an outstanding biomarker (Figure 7B), and we noted a significant positive correlation between gene and protein expression for TUBB3, with an R value of 0.67 (*p*<0.0001). However, as we reported previously [16], its predictive role was mainly confined to the HuR C pattern (Table 2). Patients with high levels of TUBB3 (i.e., from gene or protein) exhibited early relapse and shorter survival when the pattern of staining was HuR C. This association underscores the importance of the HuR-mediated TUBB3 enhancement of translation in the cytoplasm. We analyzed the data taking into consideration the expression levels of miR-200c. In keeping with the hypothesis derived from in vitro experiments, patients exhibiting high expression levels of TUBB3, miR-200c, and HuR C emerged with the worst outcome, while patients in whom low expression of TUBB3, high miR-200c and HuR N were found exhibited the best prognosis (Figure 8A-B). In the first category, the 5-year survival rate was only 6.7 percent, while in the second it was 52 percent (Table 3). In order to link clinical features with TUBB3 expression, we analyzed TUBB3 protein levels according to the expression of miR-200c and the HuR staining pattern (Figure 8 C-D). In patients exhibiting the HuR N pattern and high miR-200c, we detected the lowest TUBB3 expression, while in patients with high miR-200c and the HuR C pattern, we found the highest levels of TUBB3 expression; the difference was statistically significant (*p* value < 0.0001). These findings from our translational study clearly demonstrate that the predictive activity of miR-200c is strongly influenced by HuR.

**Table 1:** Clinical features of the analyzed setting of ovarian cancer patients

**Characteristics**	**No. pts (%)**
All cases	220
Age, yrs	
Median	58.5
FIGO Stage	
I-lI	29 (13.1)
III	163 (74.1)
IV	28 (12.8)
Histotype	
Papillary-serous	161 (73.2)
Mucinous	5 (2.2)
Endomietrioid	28 (12.7)
Clear Cell	13 (5.9)
Undifferentiated	10 (4.5)
Adenocarcinoma (not specified)	3 (1.4)
Ca 125	
Median (range)	625 U/mL (12.5-10000)
Status	
Dead	139 (63.1)
Alive	81(36.9)
Median follow up (Alive)	71 months

**Table 2:** Survival rates of different groups of patients

	**Alive**	**Dead**	**Survival Rate**
HuR C- TUBB3+ miR-200c-	10	21	32.2%
HuR C- TUBB3+ miR-200c+	1	14	6.7%
HuR C- TUBB3- miR-200c-	13	15	46.4%
HuR C- TUBB3- miR-200c+	4	14	22.2%
HuR N- TUBB3+ miR-200c-	9	14	39.1%
HuR N- TUBB3+ miR-200c +	3	10	23.0%
HuR N- TUBB3- miR-200c-	15	22	40.5%
HuR N- TUBB3- miR-200c +	11	10	52.0%

**Table 3:** Survival rates at 5 years and Odds Ratio (OR) for different groups of patients obtained through COX regression multivariate model

	**Alive**	**Dead**	**Survival Rate**	**OR**	**CI (0.05-0.95)**
HuR C- TUBB3+ miR-200c +	1	14	6.70%	2.02	1.11–3.62
HuR C- TUBB3- miR-200c +	4	14	22.20%	0.92	0.48–1.73
HuR N- TUBB3+ miR-200c +	3	10	23.00%	1.87	0.84–3.18
HuR C- TUBB3+ miR-200c -	10	21	32.20%	1.46	0.75–2.74
HuR N- TUBB3+ miR-200c -	9	14	39.10%	1.24	0.52–2.63
HuR N- TUBB3- miR-200c -	15	22	40.50%	1	
HuR C- TUBB3- miR-200c -	13	15	46.40%	1.35	0.59–2.53
HuR N- TUBB3- miR-200c +	11	10	52.00%	1	0.541–1.82

## Discussion

**Figure 9:**
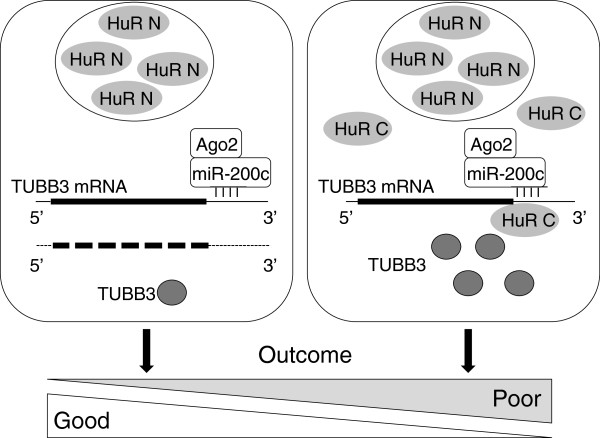
**Model proposing different mechanisms of miR-200c regulatory activity on TUBB3 mRNA in ovarian cancer.** When HuR is confined in the nucleus (HuR N), miR-200c behaves as a repressor, mediating TUBB3 mRNA decay (right panel). The resulting low levels of TUBB3 protein expression is linked to a good response to chemotherapy and a favorable outcome. On the contrary, if HuR is expressed in the cytoplasm (HuR C), the interaction with miR-200c-RISC on TUBB3 mRNA induces specific pickup of TUBB3 gene into ribosomes and enhancement of mRNA stability. This will yield up-regulation of TUBB3 protein, drug-resistance and early relapse with a poor outcome.

Ovarian cancer is a very heterogeneous disease. Tumors that underwent the epithelial to mesenchymal transition (EMT) are in general less differentiated and more invasive, aggressive, and chemoresistant. Nevertheless, a paradox appears at molecular level in ovarian cancer patients: although indicated as suppressors of EMT, enhanced expression of the miR-200 family was associated with early relapse and decreased overall survival [3-5]. In keeping with these findings, our results indicated a direct correlation between miR-200c expression and chemoresistance to paclitaxel and cisplatin in a panel of ovarian adenocarcinoma cell lines with inherent or acquired drug-resistance. In apparent contrast with these findings, in previous reports miR-200c was capable of sensitizing Hey and HeC50 cells to the effects of chemotherapy through downregulation of the TUBB3 gene [12,14]. In the A2780 model we employed, luciferase reporter assays and exogenous overexpression confirmed this dynamic and established that miR-200c acts as a negative regulator of 3^′^UTR TUBB3 mRNA. Along with TUBB3 downregulation, in this study we reported that the level of resistance to paclitaxel and cisplatin was associated with the expression level of miR-200c in A2780. Taken together, these findings suggest that miR-200c is capable of suppressing TUBB3 expression, as reported previously by Cochrane and colleagues [12,14]. However, this phenomenon is not general and in some cell lines such regulation is not detectable; in fact, exactly the opposite dynamic was observed. In cells with high TUBB3 expression, either with inherent or acquired resistance to paclitaxel/cisplatin, miR-200c expression was enhanced. How is it possible to reconcile these seemingly contradictory findings? Under specific cellular conditions, miRNA-mediated repression was prevented or reversed, and the inhibitory effect of miRNAs was modulated by RBPs acting on the same mRNA. Among the RBPs that antagonize or facilitate miRNA-mediated repression, a prominent factor is HuR, which affects stability and the translation of numerous genes implicated in cancer aggressiveness [19], including TUBB3 [16]. The ability of HuR to modulate TUBB3 mRNA expression is closely linked to its translocation into the cytoplasm, since it is dependent on the direct enhancement of translation of TUBB3 under stressing conditions. Using an S1-tagged RNP purification strategy, we discovered that, in A2780 cells, the miR-200c complex binds to the 3^′^UTR region in hypoglycemic conditions, and that such interaction increases HuR binding. At variance with the inverse relationship between miRNA expression and downregulation of this target, miRNA-mediated binding to HuR is capable of stimulating protein expression. This phenomenon appeared in vitro in some cell lines, but most importantly it also appeared in a clinical setting in which material from 220 ovarian cancer patients was analyzed. When the HuR pattern of staining was only nuclear, the prevalent mechanism by which miR-200c exerted its influence was a suppression of TUBB3 gene/protein expression, and as a consequence the tumor exhibited a good outcome. In contrast, when HuR localization was also cytoplasmic, the role of miR-200c appeared to be just the opposite, i.e., the stimulation of TUBB3 protein expression. In the latter case, we theorized that miR-200c promoted interaction with HuR in the cytoplasm and increased the translation of TUBB3, as summarized in the model of Figure 9. To our knowledge this is the first study that showed the same miRNA was capable of promoting opposite effects on the expression of one of its targets, depending on its interaction with an RBP such as HuR. This information could be valuable from a clinical perspective, because ovarian cancer is generally an aggressive disease diagnosed at an advanced stage, in which average 5-and 10-year survival in the US is 33% and 22%, respectively (SEER database, http://seer.cancer.gov). In this report, we demonstrated that the same trends observed in cell lines are detectable in patients. A good outcome, better than average, can be predicted for those patients who exhibit ovarian cancer with nuclear HuR pattern, high levels of miR-200c, and low levels of TUBB3. On the other hand, patients with HuR cytoplasmic staining and high expression of TUBB3 and miR-200c have the worst outcomes and the highest risk of death. This finding reveals the importance of multidimensional analysis when assessing the impact of miRNAs on gene expression, and it explains the discrepancies reported in the literature that analyzes the expression of miR-200c alone, claiming it as a marker of both poor [3-5] and good outcome [6-9]. We conclude that only the combined assessment of miR-200c, HuR, and TUBB3 is useful for tailoring specific protocols aimed at differentiating appropriate treatment modalities in ovarian cancer.

## Conclusions

In summary, the results of our study revealed that HuR plays a pivotal role in driving TUBB3 expression and the aggressive phenotype in ovarian cancer, through its interaction with miR-200c. We are confident that this discovery will foster the development of protocols specific to ovarian cancer patients. These findings suggest the complex HuR/miR-200c/TUBB3 as a new potential target for the development of therapies aimed at overcoming aggressive disease in ovarian cancer patients.

## Abbreviations

miRNA: microRNA; FFPE: Formalin-Fixed, Paraffin-Embedded; q-PCR: Quantitative real-time reverse transcription polymerase chain reaction; HPRT: Hypoxanthine phosphoribosyltransferase 1; RBP: RNA-binding Protein; EMT: Epithelial to Mesenchymal Transition.

## Competing interests

The authors declare that they have no competing interests.

## Authors’ contributions

SP contributed to the conception and the design of the study, performed the majority of molecular biological experiments and drafted the first version of the manuscript. GS and GF were involved in the recruitment of the patients and collected the clinical information and the informed consent. ShS collaborated in the setting of the clinical database. EM carried out the immunohistochemistry. StS performed the score of immunohistochemistry. MM analyzed TUBB3 mRNA and miR-200c expression in cancer samples. GR was involved in the design of the constructs. CF designed and coordinated the study, performed the statistical analysis and wrote the final manuscript. All the authors read and approved the final manuscript.

## Pre-publication history

The pre-publication history for this paper can be accessed here:

http://www.biomedcentral.com/1471-2407/13/72/prepub
